# Application of machine learning algorithms to predict heat-sensitive angina (HSA) attacks: a multicentric observational cohort study

**DOI:** 10.3389/fdgth.2026.1839307

**Published:** 2026-07-21

**Authors:** Jincheng Wang, Conghui Zhou, Yue Zhao, Jingqing Hu

**Affiliations:** 1 Institute of Chinese Medicine Literature, Nanjing University of Chinese Medicine, Nanjing, China; 2 Affiliated Hospital of Integrated Traditional Chinese and Western Medicine, Nanjing University of Chinese Medicine, Nanjing, China; 3 College of Traditional Chinese Medicine, Chongqing University of Chinese Medicine, Chongqing, China; 4 Chinese Medical College, Tianjin University of Traditional Chinese Medicine, Tianjin, China

**Keywords:** angina, heat-Sensitive angina (HSA), machine learning, *post hoc* analysis, risk prediction

## Abstract

**Background:**

The association between air temperature and angina has been confirmed by several studies, which show that some patients with cardiovascular disease are “heat sensitive” and experience angina more frequently in high-temperature environments. Although several predictive models for cardiovascular risk stratification and angina-related outcomes have been reported, predictive tools specifically designed to identify susceptibility to heat-sensitive angina (HSA) exacerbation under hot weather conditions remain limited.

**Methods:**

The derivation cohort consisted of 1,246 individuals with stable angina treated at 43 clinical research centers in China. Variable selection was performed using the Boruta algorithm. Seven machine learning algorithms were developed and evaluated using the area under the receiver operating characteristic curve (AUC), sensitivity, specificity, F1 score, calibration analysis, and decision curve analysis. An independent external validation cohort comprising 120 patients from 5 additional clinical research centers was used to assess model generalizability. Furthermore, we conducted a *post hoc* analysis of a multi-center clinical trial to validate the value of the model in guiding clinical strategies.

**Results:**

Through variable selection, 14 predictors were included as the risk factors to develop ML model. A random forest (RF) model demonstrated strong performance during the training and internal validation cohorts. External validation further supported the RF model's predictive performance and robustness. Exploratory *post hoc* analysis suggested that patients identified by the RF model as having high HSA probability exhibited increased angina frequency during summer months.

**Conclusions:**

This study innovatively employs region, MPA, DBP, BMI, constipation, body fat distribution indicators, and seven serological markers related to inflammation, lipid metabolism to construct an RF model. The proposed RF model demonstrated promising predictive performance for identifying patients with potential susceptibility to HSA. The model may serve as an exploratory decision-support tool for individualized heat-related cardiovascular risk assessment, although further prospective multicenter validation is required before routine clinical application.

**Clinical Trial Registration:**

https://www.chictr.org.cn/showproj.html?proj=166685, identifier ChiCTR2200060267 and https://clinicaltrials.gov/study/NCT02967718?term=NCT02967718&rank=1, identifier NCT02967718.

## Introduction

Stable angina is a common disorder worldwide with a continuously increasing prevalence (1, 2). Minimizing or eradicating symptoms of angina is the key aim of stable angina pectoris treatment (3). Angina occurs when myocardial oxygen supply does not meet myocardial demand, typically triggered by exertion or emotional stress (4). Emerging evidence suggests environmental thermal stress may constitute an underrecognized trigger (5–9). While cold-induced coronary vasoconstriction has been well documented, recent studies reveal high temperatures may exacerbate angina through multiple mechanisms, such as hemodynamic overload, dehydration-thrombosis cascade and electrolyte-mediated instability. Thermoregulated cutaneous vasodilation redistributes large amounts of cardiac output to the peripheral circulation, forcing compensatory increases in heart rate and myocardial oxygen consumption (10). This creates a “double burden” scenario in which coronary perfusion pressure decreases while myocardial oxygen demand simultaneously increases, thereby exacerbating the supply–demand mismatch (11). Profuse sweating induces plasma volume depletion, elevating hematocrit within 2 h (12). This hemoconcentration increases blood viscosity and promotes platelet aggregation via shear stress activation (13). When the temperature rises, the body sweats more to cool down, leading to the loss of electrolytes such as potassium and sodium. Sodium-potassium ATPase dysfunction during thermal stress causes intracellular K + depletion (14). Electrolyte imbalance can affect the normal functioning of the heart, causing arrhythmias and other cardiac problems that may worsen angina. Furthermore, thermal stress has been linked to an augmented risk of cardiovascular dysfunction (15), encompassing conditions such as hypertension and acute myocardial infarction. The vasodilation of peripheral blood vessels, while attempting to dissipate heat, may concurrently reduce systolic blood pressure, diminishing coronary blood flow and elevating the risk of sudden cardiac arrest (16). Additionally, Patients who use diuretics may have severe hypovolemia and consequently angina attacks or even heat shock (17).

The risk of heat-related illness results from a combination of individual susceptibility, environmental heat exposure, and other contributing factors (18). Much of the prior literature on temperature-related cardiovascular risk has focused on the effects of heat exposure on the overall patient population, with fewer studies focused on individual susceptibility and identify relevant influencing factors, like gender, ethnicity and latitude (19–22). Under a warming climate, hot weather can have an increasing impact on patients with heat-sensitive angina (HSA) pectoris, which means angina attacks will be more frequent for them in the future. More precise identification tools, preventions and interventions for patients with HSA are still lacking.

Machine learning (ML) is a new type of artificial intelligence that is beginning to be widely applied to clinical data sets for the purpose of developing robust risk models and redefining patient classes (23). In environmental epidemiology, machine learning has found numerous applications, encompassing studies on high temperature-related epidemiology, among others. For instance, Boudreault et al. developed several machine and deep learning models to predict heat-related mortality in Canada (24), while Kim et al. built a random forest (RF) model to predict CVD mortality in South Korea (25). In clinical settings, Hirano et al. built 4 ML models for heatstroke mortality predictions in Japan (26), while Fujiwara et al. used ML for heart illness detection (27). While these studies are important, none has yet used ML to predict heat-related angina attacks. The purpose of this study is to predict the risk of angina attacks in high temperature environment for patients with stable angina pectoris based on ML models.

## Methods

### Research design

**Figure 1:**
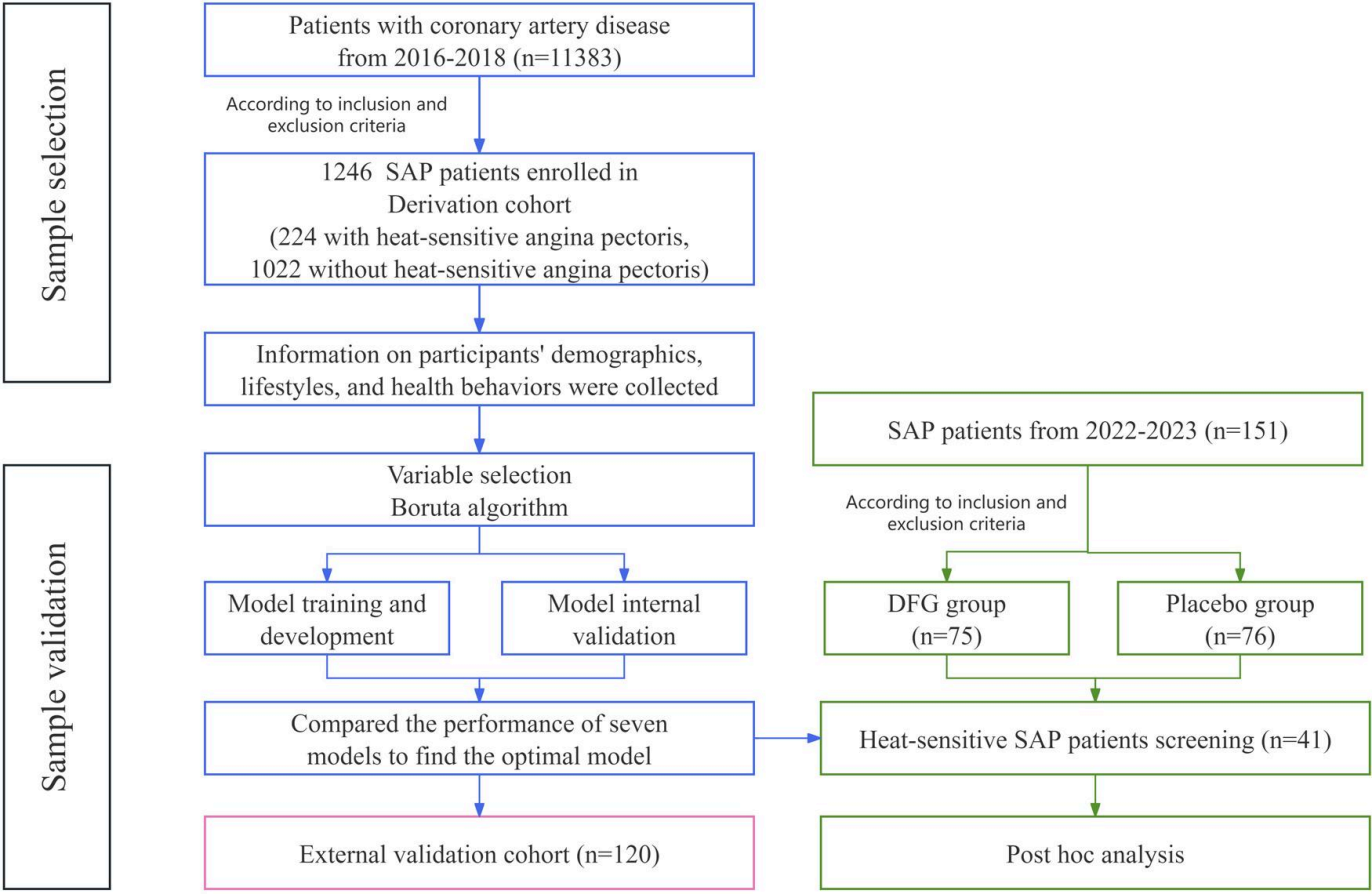
Flow diagram of the study population. SAP, stable angina pectoris; DFG, Danzhu Fuyuan Granule, a novel traditional Chinese medicine developed from the appropriate addition and reduction of Si-Miao-Yong-An decoction.

The research design for this study is depicted in Figure 1. The study was conducted in four sequential steps: (1) data collection and preprocessing; (2) feature selection and model development using the derivation cohort; (3) external validation of model performance; (4) exploratory *post hoc* subgroup analysis using an independent randomized controlled trial cohort to evaluate the potential clinical utility of the model. Initially, a training cohort, constituting 70% of the derivation cohort, was used to develop predictive models. Subsequently, the remaining 30% of the derivation cohort was designed for internal validation, while an independent validation dataset was employed for external validation. The Shapley Additive explanations (SHAP) algorithm was utilized to elucidate the significance of features in the predictive model and to identify nonlinear relationships among risk predictors. Then, we conducted a *post hoc* analysis of a nationwide, multi-center, placebo-controlled, double-blind, randomized clinical trial (RCT) regarding the use of Danzhu Fuyuan Granule (DFG) during stable angina pectoris treatment to validate the value of the model in guiding clinical strategies.

### Study subjects

The model was developed and internally validated in a population-based cohort from China, comprising over 11 000 participants aged 40–80 years at enrolment. Participants were recruited from 48 clinical research centers in 23 provinces in 5 regions (North China, Northeast China, Northwest China, Middle East China and South China) between 2016 and 2018. Supplementary Table S1 provides detailed information on clinical research centers, provinces, and regions. Cases were all outpatients or hospitalized patients with coronary artery disease. A total of three visits were conducted, with each follow-up occurring every six months. All participants underwent a baseline assessment, including a basic data collection form, physical examination, collection of blood samples and a series of questionnaires comprising the Seattle Angina Questionnaire (SAQ), the Patient Health Questionnaire (PHQ-9), and the Generalised Anxiety Disorder Questionnaire (GAD-7). This study was registered on the WHO International Clinical Trials Registry Platform (ClinicalTrials.gov) with the ID NCT02967718.

In the present study, we used all the cases of patients with stable angina pectoris [SAP, International Classification of Diseases, Eleventh Revision (ICD-11) code BA40.1] who were enrolled from the fifth week after the summer until the fall and were followed up during the winter months. Taking into account the clinical reality, the experience in the use of the SAQ scale, and the actual scores of the SAQ scale in the data of this study, the outcome variable of HSA was defined as scores on the SAQ angina frequency scale in each of the two years of follow-up that was 30 or more points lower in the summer than in the winter (28). Meteorological data for summer, and winter in the cities where the clinical research centers are located were obtained from the National Meteorological. Data Sharing Platform (http://data.cma.cn/), including daily mean, maximum and minimum temperature. These indicators are only used to determine the season and are not included in the final model. The standard for climate season delineation issued by the National Climate Center in 2012 (QX/T152-2012, https://www.cma.gov.cn/zfxxgk/gknr/flfgbz/bz/202209/t20220921_5097919.html) uses temperature thresholds of 10 ℃ and 22 ℃ to reflect climate perception and rhythms (29). For the purposes of this study, a season is defined as five consecutive days with a mean temperature sliding within a specific range: ≥22 ℃ in summer and ≤10 ℃ in winter. In the annual temperature series, the seasonal start date is determined in the following manner. The summer start date is the first date on which the five-day moving average temperature reaches or exceeds 22 ℃. The summer start date is the first date on which the five-day moving average temperature reaches or exceeds 22 ℃. The winter start date is the first day on which the five-day moving average temperature falls below 10 ℃. (If the conditions are not met after fall, the winter start date is determined from the temperature series prior to the spring start date) (30). Specific inclusion and exclusion criteria for data sources are provided in Supplementary Table S2.

The derivation cohort included patients with stable angina treated from January 2016 to December 2018 at 43 clinical research centers in Northeast China, Northwest China, Middle East China, South China and North China. A total of 3,285 individuals meeting these criteria were screened for participation. Based on the exclusion criteria, the enrollment and follow-up of the 1,735 patients did not take place during the summer or winter months, while 53 patients had incomplete or missing data. Consequently, a total of 1,246 eligible patients were selected for the derivation cohort. Among these, 224 patients had scores that met diagnostic criteria for HSA, forming the HSA group, The remaining 1,022 patients constitute non-HSA group. Then, the derivation cohort was randomly split into a ratio of 7:3 using stratified sampling, with the training cohort composed of 872 cases, of which, 158 were HSA cases, and the testing cohort containing 374 participants, including 66 individuals with HSA. Model development, feature selection, and hyperparameter tuning were performed exclusively within the training cohort. The internally validated final model was then directly applied to the external validation cohort to evaluate model generalizability. Importantly, the external validation cohort was not involved in model training, feature selection, or hyperparameter optimization at any stage.

The external validation cohort consisted of 120 stable angina patients, recruited from 5 clinical centers in five regions between January 2016 and December 2018. Among these, 26 patients had scores that met diagnostic criteria for HSA, forming the HSA group. The remaining 94 patients constituted the non-HSA group. The external validation cohort was designed to provide an independent preliminary assessment of model transportability across geographically and climatically distinct populations rather than definitive population-level validation.

### Data collection

Information on participants' demographics, lifestyles, and health behaviors were collected by uniformly trained enumerators using a group-developed information collection form. Demographic data includes sex, age, body mass index (BMI), waist, abdominal, and hip circumferences, religion, job, place of residence, moderate physical activity (MPA), smoking status (current vs. never/ former), drinking status (current vs. never/ former), systolic blood pressure (SBP), diastolic blood pressure (DBP). Laboratory tests mainly include C-reactive protein (CRP, mg/L), Homocysteine (HCY, μmol/L), HDL cholesterol (HDL-C, mmol/L), LDL cholesterol (LDL-C, mmol/L), fasting blood glucose (FBG, mmol/L), ApoA-Ⅰ (g/L), ApoB (g/L), Triglyceride (TG, mmol/L), Total Cholesterol (TC, mmol/L), uric acid (UA, μmol/L), Nitric Oxide (NO, pg/mL). The Chinese version of Patient Health Questionnaire (PHQ-9), and the Generalised Anxiety Disorder Questionnaire (GAD-7) from the website of the National Mental Health Database was used to assess depression and anxiety symptoms. The higher the score, the more severe the symptoms of depression and anxiety will be. The Chinese version of the Seattle Angina Questionnaire (SAQ) from the website of the National Center For Cardiovascular Disease was used to assess the quality of survival in patients with stable angina pectoris. The SAQ angina frequency scale range from 0 to 100 points, with higher scores indicating less angina.

### Laboratory indicator measurements

Blood samples were collected from patients on an empty stomach on the morning of the second day after enrollment. The samples were allowed to sit at room temperature for 30 min, then centrifuged at 2000×g for 20 min at 4 ℃. The serum obtained was put into the special freezing box for serum samples, stored in the refrigerator at −80 ℃, and transferred to the central laboratory for testing by centralized cold chain.

### Statistical analyses

Baseline data analysis of patients began with normality tests on the quantitative data. This study used the Kolmogorov–Smirnov test to assess the normality of data distribution for continuous variables. The data on normal distribution was represented by the mean and standard deviation and was compared using the independent samples *t*-test. Data that did not follow a normal distribution were presented using the median and interquartile range and were compared using the Mann–Whitney *U*-test. Descriptive statistics was conducted with frequencies for categorical variables presented as percentages (%) and using the chi-square or Fisher exact tests for comparison. All continuous variables underwent preprocessing prior to model training. Z-score standardization was applied to laboratory indicators (e.g., CRP, HCY) and anthropometric measures (BMI, waist circumference) to mitigate scale differences between features (31). Variables with >15% missingness were excluded; remaining missing values were imputed using multiple imputation by chained equations with predictive mean matching (5 iterations) (32). The Boruta algorithm was used to finalize the variables for inclusion, thereby eliminating any redundant features. A two-tailed *P*-value of less than 0.05 was deemed statistically significant.

The binary outcome showed a moderate class imbalance, with 250 HSA cases among 1,366 participants (18.3%). To reduce potential bias associated with class imbalance, stratified random sampling was applied during model training and internal validation to preserve the original outcome distribution across datasets. In addition, model performance was evaluated using multiple complementary metrics, including the area under the receiver operating characteristic curve (AUC), sensitivity, specificity, precision, recall, F1-score, and calibration performance, rather than relying solely on overall accuracy.

### Variable selection

The Boruta algorithm was a wrapper method for feature selection built around the Random Forest Classifier algorithm. During the model construction, Boruta created a copy of the original dataset features as Shadow Features and compared the Z-score between the actual features and shadow features calculated via Random Forest Classifier in each iteration. If the Z-score of an actual feature was higher than the maximum Z-score of shadow features, this feature was considered pivotal and kept; otherwise, it was dropped.

### Model derivation and validation

The machine learning algorithm models were developed using R software package (version 4.2.1). In this study, seven machine learning algorithms—Decision Tree (DT), Logistic Regression (Logistic), K-nearest Neighbor (KNN), Naive Bayesian (NB), light gradient boosting machine (LightGBM), Extreme Gradient Boosting (Xgboost), and Random Forest (RF)—were utilized to construct the HSA prediction model. The models were constructed as follows: The DT model using the rpart package; the Logistic model using the glm package; the KNN model using the kknn package; the NB model using the klaR package; the LightGBM model using the lightgbm package; the Xgboost model using the xgboost package; and the RF model using the randomForest package.

Each classification algorithm underwent hyperparameter tuning through 5-fold cross-validation internally. The loss function was minimized through cross-validation to identify the optimal hyperparameters. We evaluated the prediction power of each machine learning classifier using the optimal hyperparameters on the internal and external validation sets, and the performance of the models was compared using metrics such as the area under the curve, accuracy, sensitivity, specificity, and F1 score. Decision curve analysis (DCA) was performed on the testing set to evaluate the clinical utility and relative superiority of each model across different threshold probabilities. To improve model interpretability, feature importance rankings derived from the model were combined with SHAP (Shapley Additive Explanations) analysis. Feature importance reflects the relative contribution of each variable to model prediction performance but does not indicate the direction of association. Therefore, SHAP values were additionally used to quantify both the magnitude and direction of each feature's contribution to individual predictions. Positive SHAP values indicate increased predicted risk of HSA, whereas negative SHAP values indicate reduced predicted risk.

### Post hoc analyses

DFG is a novel traditional Chinese medicine developed from the appropriate addition and reduction of Si-Miao-Yong-An decoction, which has been commonly used for lipid-lowering and anti-inflammatory treatments in the clinical setting. The RCT was conducted at 7 clinical centers between May 2022 and October 2023. Inclusion and exclusion criteria were the same as those used to build the predictive model, which are provided in Supplementary Table S2. Finally, a total of 151 patients with SAP were randomly assigned in a 1:1 ratio to receive DFG (*n* = 75) or placebo (*n* = 76) at a dose of 5 g orally three times a day. Patients enrolled in the study entered a 2-week washout period followed by an 8-week period of clinical intervention. All patients underwent follow-up assessments at baseline and at 4 and 8 weeks after enrollment and were tested and evaluated at the follow-up visit. Treatment outcomes included SAQ score and nitroglycerin consumption. Specific results of the trial will be presented in a follow-up article. This study was registered in the Chinese Clinical Trial Registry and the trial registration number was ChiCTR2200060267.

Given the exploratory nature of the *post hoc* analysis, patients were classified using the RF model developed from the derivation cohort. A relatively stringent predicted probability threshold (>0.8) was selected to prioritize subgroup specificity and reduce potential false-positive classification, thereby enriching the subgroup for individuals with a high predicted likelihood of HSA. The threshold was not intended to represent a clinically validated diagnostic cut-point, but rather an exploratory enrichment strategy for hypothesis-generating subgroup analysis. Additionally, enrollment during May or June was used to ensure that follow-up assessments occurred during the summer season, when thermal exposure was most relevant to the study hypothesis. We acknowledge that these subgroup criteria were not pre-specified before trial initiation and should therefore be interpreted as exploratory and hypothesis-generating rather than confirmatory. The efficacy outcomes assessed in this subgroup analysis were scores on the SAQ angina frequency scale, CRP, GLU, TC, and LDL.

All efficacy analyses in the *post hoc* subgroup were conducted according to the intention-to-treat (ITT) principle, including all randomized participants with available follow-up data. Continuous variables were compared within groups using paired statistical tests and between groups using covariance analysis adjusted for baseline values, age, and sex. Statistical significance was defined as a two-sided *P* < 0.05.

### Ethics statement

This prediction model construction study was approved by the institute of Basic Theory for Chinese Medicine China Academy of Chinese Medical Sciences Medical Ethics Committee (No.2016EC_KY_001), and written informed consent was obtained from all participants. The randomized controlled trial study was approved by the institute of Basic Theory for Chinese Medicine China Academy of Chinese Medical Sciences Medical Ethics Committee (No.2022-KY-EC-001).

The present machine learning analysis was additionally registered at ClinicalTrials.gov (NCT02967718), and the randomized controlled trial was registered in the Chinese Clinical Trial Registry (ChiCTR2200060267).

### Role of the funding source

The funding source had no role in the study design, data collection, analysis, interpretation of data, the writing of the report, or in the decision to submit the paper for publication. All authors had full access to all data in the study and had final responsibility for the decision to submit for publication.

## Results

The study results are presented according to three distinct datasets: (1) the derivation cohort used for model development and internal validation, (2) an independent external validation cohort used to assess model generalizability, and (3) an independent randomized controlled trial cohort used exclusively for exploratory *post hoc* clinical utility analysis.

### Patient characteristics

Across the derivation and external validation cohorts, a total of 1,366 participants were included in the present analysis, including 250 patients (18.3%) classified as HSA cases and 1,116 (81.7%) classified as non-HSA cases. The comparison focused on their baseline characteristics including age at first admission, sex, marital status, education level, anxiety and depression level, BMI, waist hip ratio, life style, and the baseline levels of 8 serum indicators (Table 1). The median age was 65 years, with 56% being male. Compared with those in the Non-HSA group, people in the HSA group were more likely to reside in Eastern and Southern China, report current smoking status, engage in more weekly exercise hours, and exhibit higher waist hip ratios, FBG levels, and UA concentrations.

**Table 1:** Baseline characteristics of All participants.

Characteristic	Overall	Non-HSA group	HSA group	*p*-value
Participants, *n*	1.366	1,116	250	
Age, median (IQR), y	65.00 (59.00–72.00)	65.00 (58.00–72.25)	64.00 (61.00–69.00)	0.670
Gender, *n* (%)				0.107
Male	770 (56.37)	641 (57.44)	129 (51.60)	
Female	596 (43.63)	475 (42.56)	121 (48.40)	
Region, *n* (%)				0.003
North CN	151 (11.05)	129 (11.56)	22 (8.80)	
Northeast CN	286 (20.94)	222 (19.89)	64 (25.60)	
Northwest CN	175 (12.81)	131 (11.74)	44 (17.60)	
Middle East CN	481 (35.21)	413 (37.01)	68 (27.20)	
South CN	273 (19.99)	221 (19.80)	52 (20.80)	
Marital status, *n* (%)				0.017
Married/cohabiting	1,231 (90.12)	995 (89.16)	236 (94.40)	
Widowed/never married/divorced	135 (9.88)	121 (10.84)	14 (5.60)	
Education level, *n* (%)				0.803
Middle school and below	761 (55.71)	624 (55.91)	137 (54.80)	
High school and above	605 (44.29)	492 (44.09)	113 (45.20)	
MPA, d/wk	4.00 (2.00–7.00)	4.00 (2.00–7.00)	5.00 (2.00–7.00)	0.002
Drinking, *n* (%)				0.314
No	1,167 (85.43)	959 (85.93)	208 (83.20)	
Yes	199 (14.57)	157 (14.07)	42 (16.80)	
Smoking, *n* (%)				0.001
No	1,165 (85.29)	967 (86.65)	198 (79.20)	
Yes	201 (14.71)	149 (13.35)	52 (20.80)	
Abdominal circumference, cm	90.00 (85.00–96.00)	90.00 (85.00–95.00)	90.00 (84.00–98.00)	0.443
Waist hip ratio	0.90 (0.88–0.96)	0.89 (0.88–0.95)	1.00 (0.89–1.00)	<0.001
Pulse rate	72.00 (67.00–78.00)	72.00 (67.00–78.00)	72.00 (68.00–77.00)	0.749
SBP	132.00 (125.00–140.00)	132.00 (124.00–140.00)	132.00 (126.00–140.00)	0.452
DBP	80.00 (74.00–87.00)	80.00 (72.00–87.00)	80.00 (75.00–86.75)	0.762
Depression, *n* (%)				0.173
None—minimal	903 (66.11)	734 (65.77)	169 (67.60)	
Mild	353 (25.84)	284 (25.45)	69 (27.60)	
Moderate	80 (5.86)	70 (6.27)	10 (4.00)	
Moderately severe	30 (2.20)	28 (2.51)	2 (0.80)	
Severe	0 (0)	0 (0)	0 (0)	
Anxiety disorder, *n* (%)				0.977
None—minimal	1,107 (81.04)	902 (80.82)	205 (82.00)	
Mild	196 (14.35)	161 (14.43)	35 (14.00)	
Moderate	51 (3.73)	43 (3.85)	8 (3.20)	
Severe	12 (0.88)	10 (0.90)	2 (0.80)	
BMI, Kg/m^2^	24.62 (22.82–26.73)	24.66 (22.77–26.68)	24.48 (22.85–26.95)	0.906
FBG, mmol/L	5.38 (5.26–5.60)	5.22 (5.19–5.54)	5.60 (5.38–5.79)	<0.001
HDL, mmol/L	1.11 (0.98–1.28)	1.07 (0.99–1.29)	1.10 (0.98–1.23)	0.512
LDL, mmol/L	2.43 (1.90–3.00)	2.38 (2.04–2.96)	2.36 (1.84–2.94)	0.117
CRP, mg/L	1.02 (0.07–3.02)	1.30 (1.02–2.50)	1.50 (0.06–3.05)	0.108
TC, mmol/L	4.30 (3.62–4.96)	4.27 (3.80–4.94)	4.28 (3.76–4.80)	0.593
TG, mmol/L	1.45 (1.13–1.82)	1.47 (1.16–1.85)	1.48 (1.19–1.90)	0.257
UA, μmol/L	325.00 (296.00–362.50)	318.00 (289.50–355.00)	335.00 (325.00–341.00)	0.021
ApoA-I/ApoB	1.52 (1.31–1.71)	1.40 (1.32–1.65)	1.53 (1.52–1.56)	0.414

Data are expressed as *n* (%) or median (interauartile range), *P* values are obtained from Mann–Whitney *U*-test or chi-square test.

MPA, indicates moderate physical activity; BMI, body mass index; FBG, fasting blood glucose; HDL, high-density lipoprotein cholesterol; LDL, low-density lipoprotein cholesterol; CRP, C-reactive protein; TC, total cholesterol; TG, triglyceride; UA, uric acid; ApoA-I, Apolipoprotein A-I; ApoB, Apolipoprotein B.

### Variable selection

**Figure 2:**
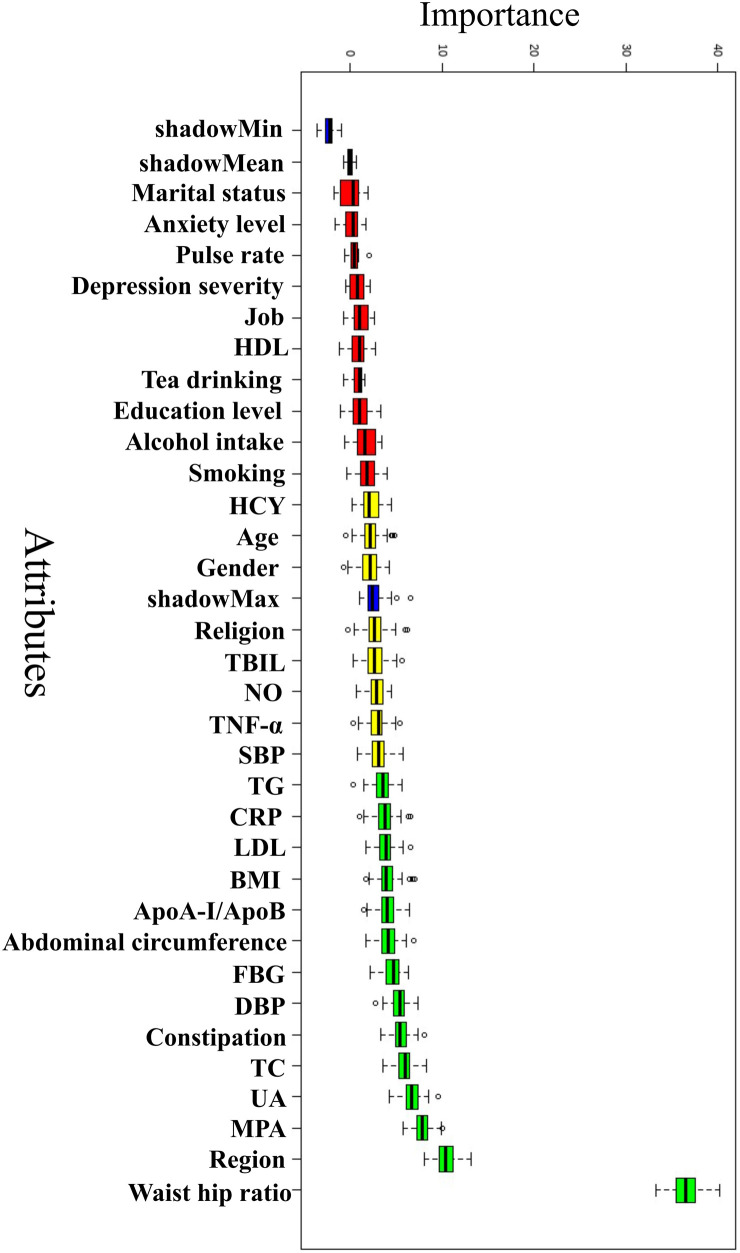
Predictor selection using Boruta. HDL, high-density lipoprotein cholesterol; HCY, homocysteine; TBIL, total bilirubin; NO, Nitric Oxide; TNF-α, tumor necrosis factor-alpha; SBP, systolic blood pressure; DBP, diastolic blood pressure; TG, triglyceride; CRP, C-reactive protein; LDL, low-density lipoprotein cholesterol; BMI, body mass index; ApoA-I, Apolipoprotein A-I; ApoB, Apolipoprotein B; FBG, fasting blood glucose; TC, total cholesterol; UA, uric acid; MPA, moderate physical activity.

Based on previous clinical work, theoretical analysis, and expert consultation, we initially identified 32 predictors: age; gender; region; marital status; education; religion; job; MPA; smoking; alcohol intake; tea drinking; anxiety level; depression severity; abdominal circumference, waist hip ratio, pulse rate, SBP, DBP, BMI, constipation, CRP, TNF-α, FBG, LDL, TC, TG, ApoA-I/ApoB, HDL, UA, HCY, TBIL, and NO. Boruta was then used for further feature selection. Boruta selected 14 predictors: region, MPA, DBP, constipation, BMI, abdominal circumference, waist hip ratio, CRP, FBG, LDL, TC, TG, UA, and ApoA-I/ApoB. The results are shown in Figure 2. The 14 selected predictors were subsequently incorporated into the machine learning models for classification of patients with stable angina pectoris as HSA or non-HSA.

### Development and evaluation of the HSA diagnostic model

**Figure 3:**
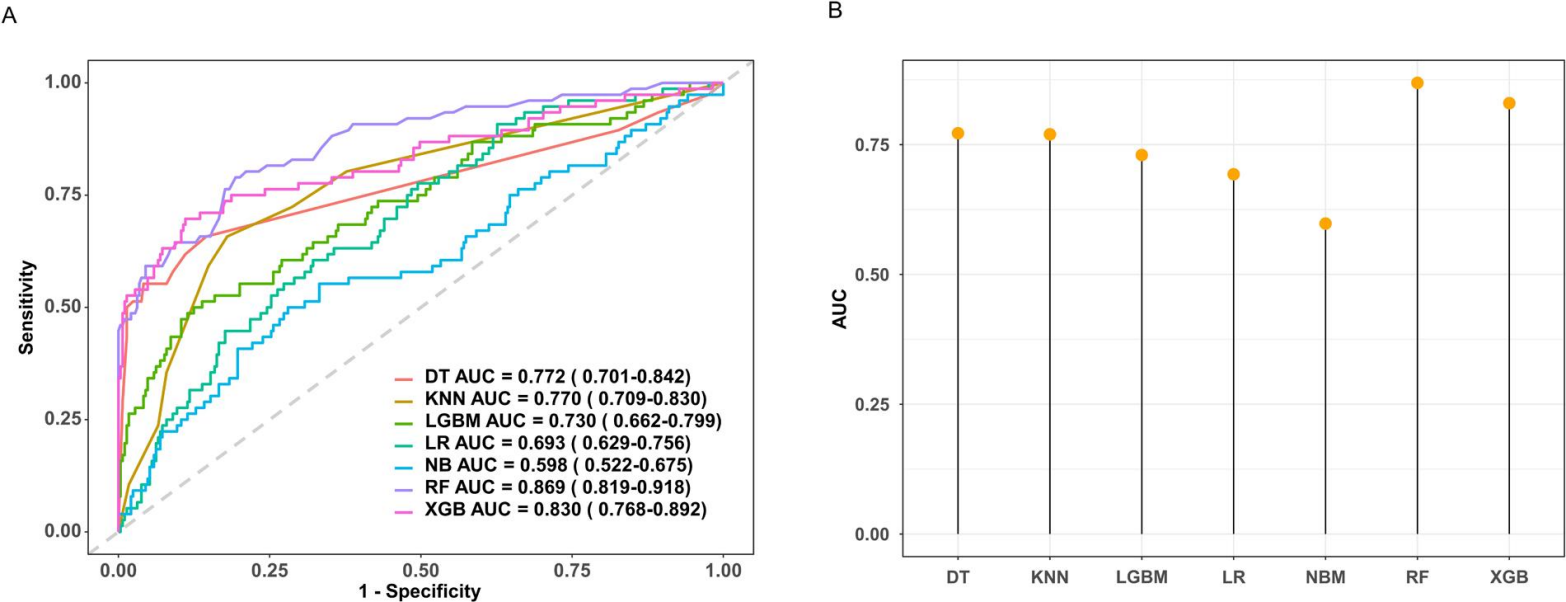
Performance comparison of seven models in the internal validation cohort. **(A)** Receiver operating characteristic (ROC) curves for each model in the internal validation cohort; **(B)** Lollipop Chart for area under the ROC curve (AUC) values with 95% confidence intervals for each model in the internal validation cohort. DT, Decision tree model; KNN, K-Nearest Neighbor model; LGBM, Light Gradient Boosting Machine model; LR, Logistic regression model; NB, Naive Bayes model; RF, Random forest model; XGB, Extreme gradient boosting model.

**Figure 4:**
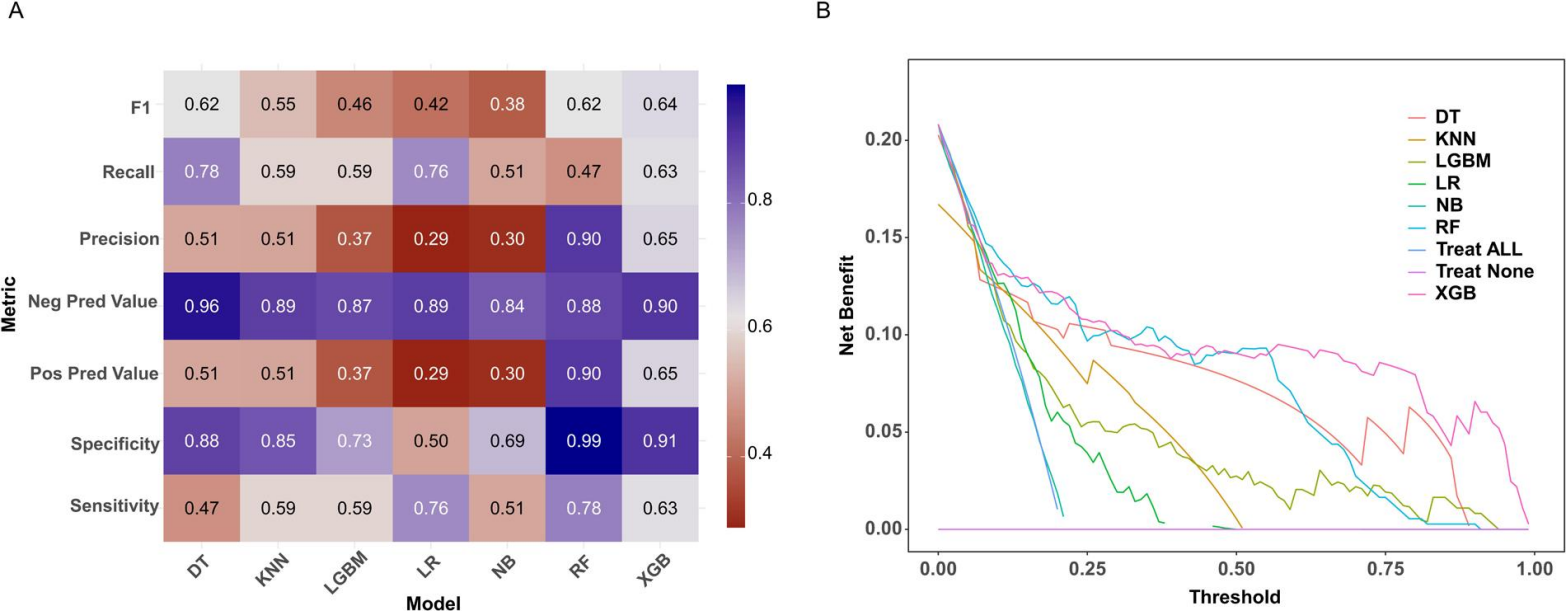
Comparative performance analysis of seven models in the internal validation cohort. **(A)** Multi-metric performance summary of each model for HSA in the internal validation cohort; **(B)** Decision curve analysis (DCA) curves for each model in the internal validation cohort. HSA, heat-sensitive angina; DT, Decision tree model; KNN, K-Nearest Neighbor model; LGBM, Light Gradient Boosting Machine model; LR, Logistic regression model; NB, Naive Bayes model; RF, Random forest model; XGB, Extreme gradient boosting model.

In the model training, a positive class represented the presence of HSA, while a negative class represented the absence of HSA. Following variable selection, the input data to train the model consisted of serum levels for 7 indicators closely related to the mechanism of angina pectoris, in addition to region, MPA, DBP, constipation, BMI, abdominal circumference and waist hip ratio of patients at admission. Utilizing these 14 features, we developed seven different machine learning models, including DT, Logistic, KNN, NB, LGBM, Xgboost, and RF. Among all evaluated machine learning algorithms, the RF model achieved the highest AUC in the internal validation cohort (Figure 3). Further examination of the data in the internal validation cohort revealed that the RF model exhibited an accuracy of 0.90, a sensitivity of 0.78, a specificity of 0.99, a positive predictive value of 0.90, a negative predictive value of 0.88, an F1 score of 0.62, and an AUC value of 0.87 (Figure 4A and Table 2). Overall, the RF model demonstrated superior predictive performance across multiple evaluation metrics. The DCA results (Figure 4B) indicated that the RF model provided the greatest net benefit across a range of threshold probabilities.

**Table 2:** Diagnostic performance of each model for HSA in internal validation cohort.

**Model**	**Sensitivity**	**Specificity**	**Pos Pred Value**	**Neg Pred Value**	**Precision**	**Recall**	**F1**	**Prevalence**	**Detection Rate**	**Detection Prevalence**	**Accuracy**
LR	0.70	0.56	0.28	0.89	0.28	0.70	0.40	0.20	0.13	0.49	0.63
DT	0.82	0.90	0.48	0.98	0.48	0.82	0.61	0.10	0.09	0.18	0.86
RF	0.55	0.93	0.63	0.91	0.63	0.55	0.58	0.18	0.10	0.15	0.74
XGB	0.83	0.84	0.55	0.95	0.55	0.83	0.66	0.19	0.16	0.29	0.84
KNN	0.70	0.51	0.23	0.89	0.23	0.70	0.35	0.18	0.12	0.53	0.61
LGBM	0.77	0.74	0.39	0.94	0.39	0.77	0.51	0.18	0.14	0.35	0.75
NB	0.53	0.76	0.32	0.88	0.32	0.53	0.40	0.18	0.09	0.29	0.65

HSA, heat-sensitive angina; LR, Logistic regression model; DT, Decision tree model; RF, Random forest model; XGB, Extreme gradient boosting model; KNN, K-Nearest Neighbor model; LGBM, Light Gradient Boosting Machine model; NB, Naive Bayes model.

### External validation of the HSA diagnostic model

**Figure 5:**
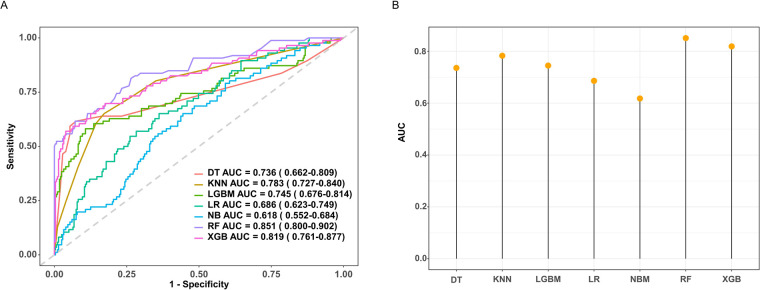
Performance comparison of seven models in the external validation cohort. **(A)** Receiver operating characteristic (ROC) curves for each model in the external validation cohort; **(B)** Lollipop Chart for area under the ROC curve (AUC) values with 95% confidence intervals for each model in the external validation cohort. DT, Decision tree model; KNN, K-Nearest Neighbor model; LGBM, Light Gradient Boosting Machine model; LR, Logistic regression model; NBM, Naive Bayes model; RF, Random forest model; XGB, Extreme gradient boosting model.

**Figure 6:**
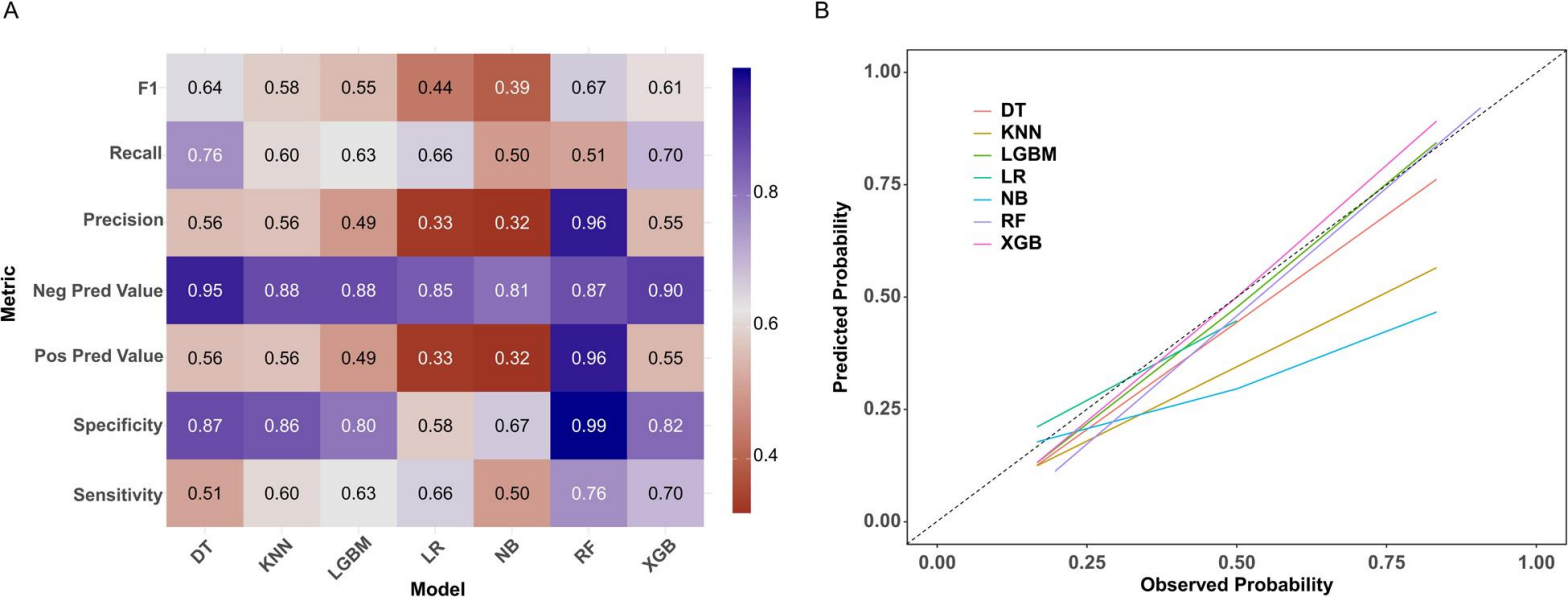
Comparative performance analysis of seven models in the external validation cohort. **(A)** Multi-metric performance summary of each model for HSA in the internal validation cohort; **(B)** Calibration characteristics analysis for each model in the external validation cohort. HSA, heat-sensitive angina; DT, Decision tree model; KNN, K-Nearest Neighbor model; LGBM, Light Gradient Boosting Machine model; LR, Logistic regression model; NBM, Naive Bayes model; RF, Random forest model; XGB, Extreme gradient boosting model.

This study involved conducting an external validation and performance comparison of seven machine learning models using a cohort of 120 stable angina patients from 5 clinical research centers in different regions. In terms of predictive performance, the results obtained from the external validation cohort indicated that the RF model achieved an AUC of 0.85, followed by the Xgboost model with an AUC of 0.82, the KNN model with an AUC of 0.78, the LGBM model with an AUC of 0.75, the DT model with an AUC of 0.74, the Logistic model with an AUC of 0.69, and the NB model with an AUC of 0.62, as depicted in Figure 5. Furthermore, Table 3 provides an overview of the accuracy, sensitivity, specificity, positive predictive value，negative predictive value and F1 Score of the seven models in the external validation cohort. The RF model exhibited an accuracy of 0.96, a sensitivity of 0.76, a specificity of 0.99, a positive predictive value of 0.96, a negative predictive value of 0.87, and an F1 score of 0.67 (Figure 6A and Table 3). Data visualization was performed to assess the predictive performance of the seven models on the external validation cohort. The calibration curve (Figure 6B) demonstrated satisfactory agreement between the RF model's predicted risk and the observed risk. Overall, the external validation results further supported the robustness and predictive performance of the RF model for identifying patients at increased risk of HSA.

**Table 3:** Diagnostic performance of each model for HSA in external validation cohort.

**Model**	**Sensitivity**	**Specificity**	**Pos Pred Value**	**Neg Pred Value**	**Precision**	**Recall**	**F1**	**AUC**	**Accuracy**
LR	0.66	0.58	0.33	0.85	0.33	0.66	0.44	0.69	0.62
DT	0.51	0.87	0.56	0.95	0.56	0.76	0.64	0.74	0.82
RF	0.76	0.99	0.96	0.87	0.96	0.51	0.67	0.85	0.75
XGB	0.70	0.82	0.55	0.90	0.55	0.70	0.62	0.82	0.76
KNN	0.61	0.86	0.57	0.88	0.57	0.61	0.58	0.78	0.73
LGBM	0.63	0.80	0.50	0.88	0.50	0.63	0.55	0.75	0.72
NB	0.50	0.67	0.32	0.81	0.32	0.50	0.39	0.62	0.59

HSA, heat-sensitive angina; LR, Logistic regression model; DT, Decision tree model; RF, Random forest model; XGB, Extreme gradient boosting model; KNN, K-Nearest Neighbor model; LGBM, Light Gradient Boosting Machine model; NB, Naive Bayes model.

### Model interpretation

**Figure 7:**
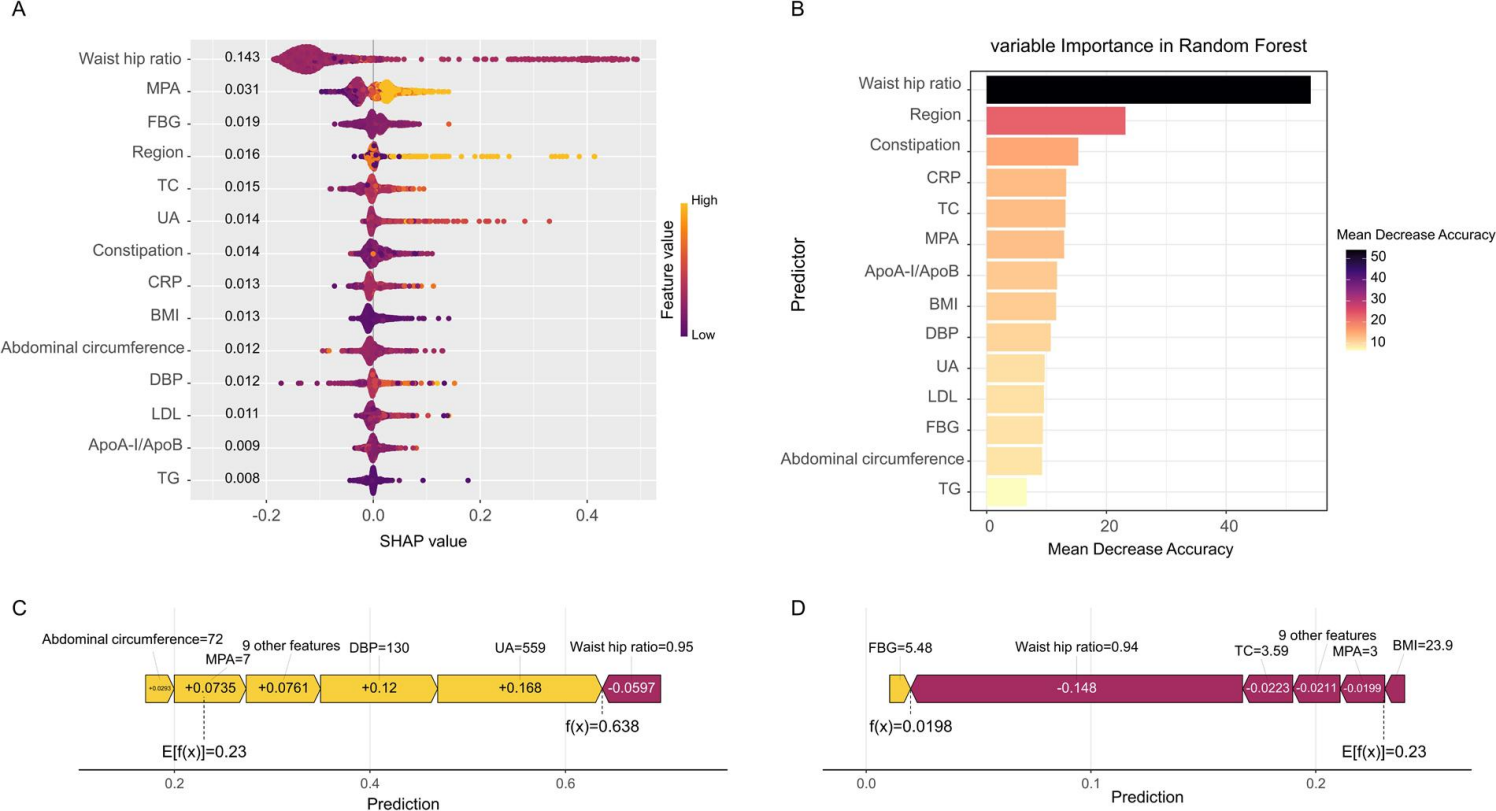
Interpretable machine learning analysis of risk factors. **(A)** All samples and features are illustrated, with each row representing a feature and *x*-axis representing the SHAP value. The purple dots represent higher feature values, while the yellow dots represent lower feature values; **(B)** Ranking of variable importance based on the average value; **(C)** SHAP predictions for HSA samples; **(D)** SHAP predictions for non-HSA samples. Yellow arrows indicate a higher risk of HSA, while purple arrows indicate a lower risk of HSA. The length of the arrows helps visualize the degree of impact of the prediction, whereby the longer the arrow, the more significant the effect. DBP, diastolic blood pressure; TG, triglyceride; CRP, C-reactive protein; LDL, low-density lipoprotein cholesterol; BMI, body mass index; ApoA-I, Apolipoprotein A-I; ApoB, Apolipoprotein B; FBG, fasting blood glucose; TC, total cholesterol; UA, uric acid; MPA, moderate physical activity.

To ensure a comprehensive understanding of the selected variables, we employed the SHAP algorithm to highlight their predictive importance in the optimal RF model for HSA. Figure 7A visually demonstrates the 14 key features of the RF model, including region, MPA, DBP, constipation, BMI, abdominal circumference, waist hip ratio, and 7 serum indicators. The influence of each feature is illustrated by uniquely colored dots: The purple dots represent higher feature values, while the yellow dots represent lower feature values. Figure 7B depicts the hierarchical organization of these 14 risk factors, underlining their significance in the model. The *x*-axis, representing SHAP values, indicates the importance of each factor. The prominent contribution of the seven serum biomarkers to model predictions suggests that these variables may play an important role in identifying patients with increased susceptibility to HSA. The SHAP analysis further illustrated how individual features influenced model predictions. Higher SHAP values corresponded to increased predicted HSA risk, whereas lower or negative SHAP values indicated reduced predicted risk. Importantly, SHAP interpretation reflects the contribution of variables to model output rather than direct causal relationships. The combination of RF feature importance and SHAP analysis therefore provided complementary information regarding both the relative influence and predictive directionality of the included variables. Additionally, we provide two typical examples, one predicting HSA (Figure 7C) and the other predicting non-HSA (Figure 7D), to demonstrate the model's interpretability.

### Post hoc analysis in the independent randomized controlled trial cohort

To further evaluate the potential clinical utility of the RF model, we performed a *post hoc* subgroup analysis using an independent randomized controlled trial (RCT) cohort that was not involved in model development or validation. Patients enrolled in the DFG trial were classified using the RF model established in the derivation cohort. Individuals with a predicted HSA probability >0.8 and enrolled during May or June were defined as the HSA subgroup.

In the *post hoc* ITT analysis of the exploratory HSA subgroup, baseline characteristics were well balanced between the DFG and placebo groups (Supplementary Table S3). Within-group comparisons showed that the SAQ angina frequency score remained stable in the DFG group, whereas a significant decline was observed in the placebo group during the summer period (*P* < 0.05). At 8 weeks after enrollment, the placebo group exhibited significantly worse SAQ angina frequency scores during summer follow-up.

In the DFG group, CRP, TC, and LDL levels showed significant reductions after treatment (all *P* < 0.05). After adjustment for baseline SAQ angina frequency score, sex, and age, between-group comparisons demonstrated statistically significant differences in SAQ angina frequency score, CRP level, and LDL level between the DFG and placebo groups (all adjusted *P* < 0.05) (Table 4).

**Table 4:** Outcomes of patients with HSA in study population for intention-to-treat analyses.

Outcomes	DFG group (*n* = 22)	Placebo group (*n* = 19)	Between-group difference	*p*-value
CRP, mg/L				
Baseline	4.04 (2.81)	4.50 (2.87)	−0.46(−0.66 to 1.74)	
Adjusted changes	−3.58 (6.31)*	1.58 (1.57)*	−5.16(−7.62 to −3.36)	<0.001
TC, mmol/L				
Baseline	5.20 (1.70)	4.76 (0.84)	0.44(−0.32 to 1.28)	
Adjusted changes	−0.85 (0.76)^#^	0.58 (0.45)	−1.43(−2.74 to 1.38)	<0.001
LDL, mmol/L				
Baseline	2.25 (0.81)	1.86 (0.51)	0.39(−0.76 to 1.95)	
Adjusted changes	−0.48 (0.37)*	0.36 (0.30)	−0.84(−1.92 to 1.65)	<0.001
SAQ angina frequency scale score				
Baseline	62.73 (12.80)	68.95 (11.00)	−6.22(−9.35 to −4.32)	
Adjusted changes	21.36 (4.60)^#^	−12.89 (3.40)*	34.25 (25.75 to 42.88)	<0.001

Baseline values are presented as mean (SD). Adjusted changes are presented as mean (SE). Between-group differences are presented as mean differences with 95% confidence intervals. Models were adjusted for baseline SAQ angina frequency score, sex, and age as fixed effects, with patient included as a random effect, and randomization group included as a class variable for between-group comparisons.

* *p* ≤ 0.05.

# *p* ≤ 0.001.

HSA, heat-sensitive angina; DFG, Danzhu Fuyuan Granule; SAQ, Seattle Angina Questionnaire; CRP, C-reactive protein; LDL, low-density lipoprotein cholesterol.

These exploratory findings suggest that the RF model may have potential utility for identifying patients with increased susceptibility to HSA aggravation. However, because the subgroup definition was derived from the same predictive framework, the results should be interpreted cautiously and require prospective independent validation.

## Discussion

In this multicenter retrospective study, we developed and validated a machine learning model for predicting HSA in patients with stable angina. Using Boruta feature selection and seven machine learning algorithms, we identified 14 key predictors and found that the RF model achieved the best overall predictive performance in both the internal validation and independent external validation cohorts. SHAP analysis further improved model interpretability by quantifying the contribution of individual predictors. Using this model, we identified patients at increased predicted risk of HSA and performed a *post hoc* analysis based on the factors in the model, which provided preliminary evidence supporting the potential relevance of anti-inflammatory and lipid-lowering strategies in patients with HSA, with patients in the study group scoring on average 23.04 points higher on the SAQ angina frequency dimension during the summer months than those in the placebo group.

From the point of view of influence factors, the Boruta algorithm assisted in identifying 14 significant variables including region, MPA, DBP, constipation, BMI, abdominal circumference, waist hip ratio, CRP, FBG, LDL, TC, TG, UA, and ApoA-I/ApoB. Lipid metabolism, systemic fat distribution, and inflammation emerged as major factors associated with sensitivity to high temperatures in patients with stable angina pectoris. Independent of heat exposure, these factors are well-established contributors to stable angina (33, 34). Abnormal lipid metabolism, particularly elevated levels of low-density lipoprotein cholesterol (LDL-C), total cholesterol (TC), and triglycerides, is closely related to the development of SAP (33, 35–37). Elevated LDL-C promotes atherosclerosis and reduces coronary blood flow (38). Higher TC levels are associated with more severe coronary stenosis (39). A lower ApoA-I/ApoB ratio has been significantly associated with atherosclerotic disease and SAP, as reduced ApoA-I levels increase the risk of cardiovascular events (40–42). ApoB lipoproteins that contain apoCIII increase THP-1 cell adhesion to ECs via PKC alpha and RhoA-mediated beta 1-integrin activation, may directly contribute to the development of atherosclerosis (43). In addition, oxidized LDL (ox-LDL) damages endothelial cells, triggering further inflammation and promoting plaque formation (44, 45). Statins reduce cardiovascular risk through both lipid-lowering and anti-inflammatory effects (46). Disorders of lipid metabolism frequently co-occur with obesity. Obesity is a major risk factor for SAP due to its close association with dyslipidemia, insulin resistance, and systemic inflammation (47). Adipose tissue is an active endocrine organ that secretes pro-inflammatory cytokines, such as tumor necrosis factor-alpha (TNF-α) and IL-6, which exacerbate atherosclerosis (48). Inflammation is a recognized factor in the development and progression of atherosclerosis, the underlying cause of SAP. In patients with SAP, elevated levels of inflammatory markers such as C-reactive protein (CRP) and interleukin-6 (IL-6) have been observed (49–51). CRP, in particular, is considered an independent risk factor for predicting coronary events in SAP patients, as it promotes endothelial dysfunction and accelerates the atherosclerotic process, increasing the risk of myocardial ischemia (52). The inflammatory response leads to endothelial dysfunction, impairing the coronary arteries' ability to dilate properly in response to increased oxygen demand (34). Additionally, increased macrophage infiltration into atherosclerotic plaques makes them more unstable, raising the risk of ischemic episodes during physical activity (53). Elevated FBG levels, often seen in SAP patients, further increase inflammation and oxidative stress (54, 55).

This study highlights a significant association between elevated waist hip ratio and increased susceptibility to HSA. Waist hip ratio, a robust indicator of central adiposity and visceral fat deposition (56), may contribute to HSA vulnerability through multiple interconnected physiological pathways. Visceral adipose tissue is metabolically active, releasing pro-inflammatory cytokines and free fatty acids that promote endothelial dysfunction, systemic inflammation, and insulin resistance—all established contributors to coronary microvascular dysfunction and reduced ischemic threshold (57). Crucially, these factors also impair thermoregulatory capacity (58). During heat stress, the cardiovascular system faces dual demands: increased cardiac output for thermoregulatory skin blood flow and potential coronary perfusion mismatch. Individuals with high waist hip ratio may experience amplified cardiac strain. Central obesity compromises heat dissipation by reducing effective body surface area for radiation/convection and burdening cardiac output requirements (58). Furthermore, the underlying endothelial dysfunction impairs vasodilatory responses critical for both coronary perfusion and cutaneous heat loss (57). This confluence likely lowers the angina threshold specifically under thermal stress. The findings align with population studies linking heatwaves to acute cardiovascular events, particularly in those with pre-existing metabolic dysfunction (59, 60), and underscore waist hip ratio as a key modifiable risk factor for HSA susceptibility.

Despite the substantial body of literature indicating the impact of these factors on stable angina and the lipid-lowering and anti-inflammatory therapeutic measures employed by physicians, a considerable number of patients with angina remain inadequately managed in clinical practice. These findings may contribute to a better understanding of risk stratification in patients with HSA. With respect to the isolated effect of heat, some literature has investigated the influence of heat on the entire population of patients with stable angina pectoris. High temperatures exacerbate SAP symptoms by increasing cardiovascular strain (61). Heat exposure leads to vasodilation and an increase in heart rate, raising the myocardial oxygen demand (62). Additionally, dehydration and electrolyte imbalances during heatwaves may impair coronary perfusion, increasing the likelihood of angina attacks (63). Extreme heat activates the sympathetic nervous system, placing additional strain on the heart and triggering cardiovascular events (64). Studies have shown that hospital admissions for cardiovascular conditions significantly increase during heatwaves, particularly in patients with pre-existing coronary artery disease (65). Furthermore, extreme temperatures may exacerbate endothelial dysfunction, complicating the management of SAP in summer months (66). Research indicates that mortality rates among SAP patients rise significantly during heatwaves, underscoring the need for tailored temperature management strategies for these individuals (67). However, the frequency of days with elevated temperatures is increasing as global warming progresses. Therefore, cooling measures alone may be insufficient. If prospectively validated, the model may help identify patients who could benefit from preventive strategies during hot weather. These patients can then be given anti-inflammatory and lipid-lowering medications, as well as being prescribed dietary and exercise regimens that modify fat distribution and control abdominal circumference and waist-to-hip ratio. Lifestyle modification, particularly diet and regular exercise, is essential for improving fat distribution and reducing central obesity. Aerobic exercises such as walking, cycling, and swimming have been shown to reduce visceral fat and improve metabolic outcomes (68, 69). Resistance training, when combined with aerobic exercise, enhances muscle mass and further promotes fat loss (70). A balanced diet rich in whole grains, fruits, and vegetables, and low in processed foods, supports these effects by improving insulin sensitivity and reducing inflammation (71). Recent studies suggest that high-intensity interval training (HIIT) may be particularly effective in reducing abdominal fat (72, 73). Sustainable lifestyle interventions focusing on both physical activity and nutrition play a critical role in managing obesity-related risks, including stable angina pectoris (74).

In this study, a HSA prediction model was developed using seven widely used machine learning algorithms for the processing of medical data. The RF model demonstrated superior predictive performance compared with the other evaluated algorithms in both the training and validation cohorts. DCA also indicated superior clinical utility of the RF model. External validation further supported model robustness, with the RF model achieving an AUC of 0.85. This result may partly reflect characteristics of the study population and sample size. Random forests are ensemble models composed of multiple decision trees trained on bootstrap-resampled subsets of the data. The small sample set has a weaker representation compared to the dataset as a whole, but it also makes some of the extremes more significant (75). A *post hoc* analysis of an independent multicentre randomized controlled trial cohort provided exploratory evidence supporting the potential association of fat distribution and inflammatory markers with HSA susceptibility. Following the administration of lipid-lowering and anti-inflammatory therapies, patients with HSA exhibited a 23.04-point improvement in Seattle Angina Scale frequency dimension scores during the summer months compared with controls. These findings provide preliminary evidence supporting the clinical relevance of the model. Importantly, the *post hoc* subgroup analyses were not designed to provide confirmatory evidence of clinical efficacy, but rather to explore the potential clinical relevance of the proposed prediction framework and generate hypotheses for future prospective studies.

From the perspective of model interpretation, traditional machine learning algorithms are often critiqued for their lack of transparency and interpretability. SHAP analysis improved model transparency by quantifying both the magnitude and direction of individual predictor contributions. Combined with feature-importance ranking, it provided a more interpretable explanation of model predictions.

An important consideration in the interpretation of the present model is the balance between sensitivity and specificity. In this study, a relatively stringent probability threshold was adopted during the exploratory subgroup analysis to prioritize specificity and reduce false-positive classification. This strategy was intended to enrich the subgroup for individuals with a higher predicted likelihood of HSA, although it may have reduced sensitivity and potentially excluded some at-risk patients. The optimal balance between sensitivity and specificity depends on the intended clinical application. For example, screening-oriented strategies may prioritize higher sensitivity to reduce missed high-risk individuals, whereas targeted preventive interventions may favor higher specificity to improve subgroup precision and reduce unnecessary interventions. Therefore, future prospective studies should further evaluate threshold optimization according to specific clinical objectives and healthcare settings.

Previous cardiovascular prediction models have primarily focused on major adverse cardiovascular events, mortality risk, coronary artery disease severity, or general angina-related outcomes. In contrast, the present study specifically addressed susceptibility to HSA exacerbation under hot weather conditions, a clinically relevant but comparatively underexplored dimension of cardiovascular risk. Rather than replacing established cardiovascular risk stratification frameworks, the proposed RF model may serve as a complementary tool that incorporates climate-related vulnerability into symptom-oriented cardiovascular risk assessment. This distinction may become increasingly important as heat exposure and climate-related cardiovascular risks continue to rise. In addition, compared with traditional regression-based prediction approaches, the RF model combined with SHAP analysis enabled modeling of potentially complex nonlinear interactions while also improving interpretability at the individual feature level. Nevertheless, direct comparisons with established cardiovascular prediction models were beyond the scope of the present study and should be evaluated in future investigations.

Clinically, the proposed RF model may help identify patients with stable angina who are more vulnerable to symptom aggravation during hot weather. Such individuals may benefit from closer monitoring, optimization of cardiovascular risk factors, and targeted preventive interventions. However, the model should currently be regarded as an exploratory decision-support tool that requires prospective multicenter validation before routine clinical use.

Because the model relies on routinely available demographic, clinical, and laboratory variables, it may be readily integrated into existing cardiovascular care workflows or electronic health record systems. In practice, patients identified as having increased HSA susceptibility could receive enhanced seasonal monitoring, lifestyle counseling, and heat-exposure prevention strategies. Nevertheless, implementation feasibility, cost-effectiveness, and real-world clinical impact remain to be established in prospective studies.

Although the model was developed and externally validated using multicenter cohorts from different regions of China, its performance in other ethnic, climatic, and healthcare settings remains uncertain. International multicenter validation is therefore required before broader implementation can be considered.

### Strengths and limitations

This study's primary contributions to the field can be attributed to three key areas of innovation. Firstly, the present study extends previous cardiovascular risk prediction research by focusing specifically on heat-related angina susceptibility in patients with stable angina. This study further extends the concept of stable angina by incorporating susceptibility to hot weather as a clinically relevant phenotype. Secondly, an interpretable machine learning model was constructed utilising readily accessible demographic data and serum indicators. Thirdly, exploratory *post hoc* analyses were conducted to investigate the potential clinical relevance of the model and to generate hypotheses regarding the possible role of anti-inflammatory and lipid-lowering strategies in patients with HSA.

Several limitations of this study should be acknowledged. First, although the model was developed using multicenter data from different regions of China and externally validated in an independent Chinese cohort, its transportability across broader international populations, healthcare systems, and climate environments remains uncertain. In particular, regional differences in heat stress intensity and seasonal climate variation may influence the manifestation of HSA symptoms (76, 77). While the external validation cohort provided important preliminary evidence for model transportability across climatic regions, broader international validation remains necessary.

Second, although the study included a relatively large multicenter cohort, the effective number of HSA cases remained limited, which may constrain model complexity and affect the stability of certain machine learning algorithms. In addition, the binary outcome exhibited moderate class imbalance, which may still influence model sensitivity and subgroup prediction performance despite the use of stratified sampling and multi-metric evaluation strategies. Furthermore, the external validation cohort size was relatively modest, limiting comprehensive assessment of model generalizability across diverse climate zones.

Third, although an independent external validation cohort was included, the internal validation process relied on a single 70/30 train-validation split rather than repeated cross-validation or bootstrap resampling. This approach may increase the risk of performance instability associated with random data partitioning. Future studies with larger datasets should incorporate more robust resampling-based validation strategies to further improve model stability and reproducibility.

Fourth, the *post hoc* subgroup analysis has several important methodological limitations. The HSA subgroup definition was based on model-derived probabilities and seasonal enrollment timing, which were not prospectively pre-specified. Although a relatively stringent probability threshold (>0.8) was selected to prioritize subgroup specificity and reduce false-positive classification, this data-driven strategy may introduce selection bias, reduce sensitivity, and increase the risk of overfitting. Moreover, because the same RF framework was used both to define and evaluate the subgroup, a degree of circular reasoning cannot be excluded. Therefore, these findings should be interpreted as exploratory and hypothesis-generating rather than confirmatory.

Finally, the retrospective nature of multicenter data collection inevitably resulted in a degree of missing data and potential heterogeneity in clinical assessment across centers. Although strict inclusion and exclusion criteria and standardized preprocessing procedures were applied to mitigate these issues, prospective international multicenter studies are still required to further validate the robustness, transportability, and clinical utility of the proposed model.

## Conclusions

In conclusion, this study developed and externally validated an RF-based predictive model for estimating susceptibility to HSA under hot weather conditions. The model demonstrated promising discrimination, interpretability, and consistent performance in both internal and independent external validation using routinely available clinical variables and SHAP-based analysis. These findings suggest that machine learning approaches may have potential utility in climate-related cardiovascular risk stratification and individualized seasonal symptom assessment. However, the present results should be considered exploratory and hypothesis-generating, and further prospective multicenter studies are required to confirm the robustness, generalizability, and real-world clinical utility of the proposed model.

## Data Availability

The raw data supporting the conclusions of this article will be made available by the authors, without undue reservation.
