# Supplementary material for: Application of machine learning algorithms to predict heat-sensitive angina (HSA) attacks: a multicentric observational cohort study
Source: Front Digit Health. 2026 Jul 21;8:1839307. doi: 10.3389/fdgth.2026.1839307 (PMC13435039; doi:10.3389/fdgth.2026.1839307)
Supplement: Supplementary file 1 [file Datasheet1.docx]

**SUPPLEMENTAL MATERIAL**

**Table S1. Information on clinical research centers, provinces, and regions**

| **Regions** | **Provinces** | | | **Clinical research centers** | **Encoding** | **Participants** |
| --- | --- | --- | --- | --- | --- | --- |
| North China | Beijing | | | Beijing First Hospital of Integrated Chinese and Western Medicine | 0101 | 6 |
|  |  |  |  | Beijing Fengtai Hospital of Integrated Chinese and Western Medicine | 0102 | 8 |
|  |  |  |  | Xiyuan Hospital of China Academy of Chinese Medical Sciences (CACMS) | 0104 | 2 |
|  |  |  |  | Dongfang Hospital of Beijing University of Chinese Medicine | 0105 | 5 |
|  |  |  |  | Gaung’anmen Hospital of CACMS | 0106 | 52 |
|  |  |  |  | Beijing Chaoyang District Sanmafang Hospital | 0107 | 38 |
|  | Tianjin | | | First Teaching Hospital of Tianjin University of Traditional Chinese Medicine | 0201 | 7 |
|  |  |  |  | The Second Affiliated Hospital of Tianjin University of Traditional Chinese Medicine | 0202 | 3 |
|  | Hebei | | | School of Basic Medical Sciences, Hebei University of Chinese Medicine | 0301 | 12 |
|  |  |  |  | Hebei Provincial Hospital of Traditional Chinese Medicine | 0302 | 18 |
| Northeast China | Inner Mongolia | | | The Affiliated Hospital of Inner Mongolia Medical University | 0401 | 54 |
|  |  |  |  | People's Hospital of Inner Mongolia Autonomous Region | 0402 | 15 |
|  | Heilongjiang | | | The First Hospital Affiliated to Heilongjiang University of Chinese Medicine | 0501 | 49 |
|  | Jilin | | | Affiliated Hospital of Changchun University of Traditional Chinese Medicine | 0601 | 51 |
|  | Liaoning | | | Affiliated Hospital of Liaoning University of Traditional Chinese Medicine | 0701 | 57 |
|  |  |  |  | The Second Affiliated Hospital of Liaoning University of Traditional Chinese Medicine | 0702 | 60 |
| Middle East China | Shandong | | | Affiliated Hospital of Shandong University of Traditional Chinese Medicine | 0801 | 74 |
|  |  |  |  | Central Hospital Affiliated to Shandong First Medical University | 0802 | 57 |
|  | Shanghai | | | Shuguang Hospital Affiliated to Shanghai University of Traditional Chinese Medicine | 0901 | 46 |
|  | Zhejiang | | | Zhejiang Provincial Hospital of Traditional Chinese Medicine | 1001 | 61 |
|  |  |  |  | Zhejiang Provincial Tongde Hospital | 1002 | 50 |
|  | Henan | | | The First Affiliated Hospital of Henan University of Chinese Medicine | 1101 | 93 |
|  |  |  |  | Yudong Hospital, The First Affiliated Hospital of Henan University of Chinese Medicine | 1102 | 92 |
|  | Jiangsu | | | Affiliated Hospital of Integrated Traditional Chinese and Western Medicine, Nanjing University of Chinese Medicine | 1201 | 8 |
| Northwest China | | Shaanxi | Shaanxi Provincial Hospital of Traditional Chinese Medicine | | 1301 | 5 |
|  |  | Xinjiang | Xinjiang Uygur Autonomous Region Hospital of Traditional Chinese Medicine | | 1401 | 14 |
|  |  |  | Urumqi Hospital of Traditional Chinese Medicine | | 1402 | 34 |
|  |  | Ningxia | The Affiliated TCM Hospital of Ningxia Medical University | | 1501 | 58 |
|  |  |  | Yinchuan Hospital of Traditional Chinese Medicine Affiliated to Ningxia Medical University | | 1502 | 46 |
|  |  |  | Ningxia Institute of Traditional Chinese Medicine | | 1503 | 5 |
|  |  | Shanxi | Shanxi Provincial Hospital of Traditional Chinese Medicine | | 1601 | 10 |
|  |  |  | Affiliated Hospital of Shanxi University of Chinese Medicine | | 1602 | 3 |
| South China | | Yunnan | Kunming Hospital of Traditional Chinese Medicine | | 1801 | 8 |
|  |  | Hunan | The First Hospital of Hunan University of Chinese Medicine | | 1901 | 18 |
|  |  | Hubei | Hubei Provincial Hospital of Traditional Chinese Medicine | | 2001 | 6 |
|  |  |  | Xiangyang Hospital of Traditional Chinese Medicine | | 2002 | 20 |
|  |  |  | Huangshi Hospital of Traditional Chinese Medicine | | 2003 | 10 |
|  |  |  | Shiyan Combined Traditional Chinese and Western Medicine Hospital | | 2004 | 11 |
|  |  |  | Shiyan Taihe Hospital | | 2005 | 27 |
|  |  | Guangxi | The First Affiliated Hospital of Guangxi University of Chinese Medicine | | 2101 | 23 |
|  |  |  | Ruikang Hospital affiliated to Guangxi University of Chinese Medicine (Nanning Hospital of Traditional Chinese Medicine) | | 2102 | 21 |
|  |  | Guangdong | Guangdong Hospital of Traditional Chinese Medicine | | 2201 | 12 |
|  |  |  | The First Affiliated Hospital of Guangzhou University of Chinese Medicine | | 2202 | 17 |
|  |  |  | Maoming Hospital of Traditional Chinese Medicine | | 2203 | 13 |
|  |  | Fujian | Department of Cardiology I, The Affiliated People's Hospital of Fujian University of Traditional Chinese Medicine | | 2301 | 11 |
|  |  |  | Department of Cardiology II, The Affiliated People's Hospital of Fujian University of Traditional Chinese Medicine | | 2302 | 35 |
|  |  |  | The Second People's Hospital Affiliated to Fujian University of Traditional Chinese Medicine | | 2303 | 23 |
|  |  | Jiangxi | Jiangxi Provincial Hospital of Traditional Chinese Medicine | | 2401 | 18 |

**Table S2. Inclusion and exclusion criteria**

**Inclusion criteria**

- Age 18-90 years at inclusion
- Capacity to consent
- Meets diagnostic criteria for stable angina pectoris (International Classification of Diseases, Eleventh Revision (ICD-11) code BA40.1)
- Enrollment from the fifth week after the summer until the fall, follow-up during the winter months

**Exclusion criteria**

- Pregnancy at the time of inclusion
- Those with severe arrhythmias (rapid atrial fibrillation, atrial flutter, paroxysmal ventricular tachycardia, etc.) with hemodynamic changes
- Heart valve disease (moderate lesions on one or more valves) or primary cardiomyopathy
- Patients with acute exacerbation of chronic obstructive pulmonary disease or pulmonary heart disease or respiratory failure
- Patients with combined acute and subacute stages of cerebrovascular disease
- Combination of serious primary diseases such as hematopoietic system or malignant tumors
- Patients with severe mental disorders (perceptual disorders, thought disorders)

**Table S3. The baseline Characteristics of two treatment arms**

| **Characteristics** | **ALL**  **(n=41)** | **DFG group (n=22)** | **Placebo group**  **(n=19)** | **P value** |
| --- | --- | --- | --- | --- |
| Sex |  |  |  | 0.260 |
| male | 32 (78.05%) | 19 (86.36%) | 13 (68.42%) |  |
| female | 9 (21.95%) | 3 (13.64%) | 6 (31.58%) |  |
| Age | 62.00 (55.00-65.00) | 61.50 (54.25-64.50) | 62.00 (58.00-66.00) | 0.402 |
| GLU(mmol/L) | 5.60 (5.23-6.00) | 5.58 (5.22-6.04) | 5.69 (5.24-6.98) | 0.647 |
| LDL(mmol/L) | 1.75 (1.65-3.00) | 1.92 (1.83-2.96) | 1.62 (1.55-3.52) | 0.174 |
| CRP(mg/L) | 1.87 (0.74-7.93) | 1.61 (0.62-7.27) | 1.89 (0.90-8.90) | 0.689 |
| TC(mmol/L) | 4.25 (3.55-4.99) | 4.59 (3.75-4.88) | 4.00 (3.65-4.81) | 0.130 |
| SAQ angina  frequency scale score | 70(60-70) | 70(65-80) | 65(50-70) | 0.106 |

Abbreviations: GLU: glucose; LDL: low-density lipoprotein cholesterol; CRP: C-reactive protein; TC: total cholesterol; SAQ: Seattle Angina Questionnaire

**Supplemental Methods**

**Hyperparameter Optimization Protocol**

The hyperparameter tuning process for each machine learning algorithm was conducted as follows^1,2^:

**Search Strategy**

1. Grid Search was employed for low-dimensional parameter spaces (Logistic Regression, KNN).
2. Random Search with 50 iterations used for tree-based models (RF, XGBoost, LightGBM).
3. Bayesian Optimization applied to Naive Bayes due to probabilistic parameter dependencies.

**Selection Criteria**

Optimal parameters were selected based on:

1. 5-fold CV AUROC maximization (primary criterion)
2. Early Stopping: Training halted if no AUROC improvement in 10 consecutive epochs
3. Complexity Penalty: Final model required <20% relative performance drop on validation vs training

**Table S4. Parameter Space Definitions**

| **Algorithm** | **Hyperparameters** | **Search Range** | **Tuning Method** |
| --- | --- | --- | --- |
| Logistic Regression | Penalty (L1/L2) C (regularization) | [0.001, 100] (log scale) | Grid Search (100-point log grid) |
| Random Forest | n_estimators max_depth min_samples_split | [50-500] [3-15] [2-10] | Random Search |
| Xgboost | learning_rate max_depth subsample | [0.01-0.3] [3-10] [0.6-1.0] | Bayesian (GP-EI) |
| LightGBM | num_leaves min_data_in_leaf feature_fraction | [16-256] [20-100] [0.6-1.0] | Random Search |

**References**

1.	Yang L, Shami A. On hyperparameter optimization of machine learning algorithms: Theory and practice. Neurocomputing. 2020;415:295-316. doi: 10.1016/j.neucom.2020.07.061

2.	Bergstra J, Bengio Y. Random Search for Hyper-Parameter Optimization. Journal of Machine Learning Research. 2012;13:281-305. doi: 10.1016/j.chemolab.2011.12.002
